# Feeding intolerance scoring system in very preterm and very low birth weight infants using clinical and ultrasound findings

**DOI:** 10.3389/fped.2024.1370361

**Published:** 2024-04-25

**Authors:** Evita Karianni Bermanshah Ifran, Badriul Hegar, Rinawati Rohsiswatmo, Wresti Indriatmi, Tetty Yuniarti, Najib Advani, Dewi Irawati Soeria Santoso, Marshita Masui, Hardya Gustada Hikmahrachim, Koen Huysentruyt, Yvan Vandenplas

**Affiliations:** ^1^ Department of Child Health, Faculty of Medicine Universitas Indonesia—Dr. Cipto Mangunkusumo National Central General Hospital, Jakarta, Indonesia; ^2^ Department of Dermatology and Venereology, Faculty of Medicine Universitas Indonesia—Dr. Cipto Mangunkusumo National Central General Hospital, Jakarta, Indonesia; ^3^ Department of Child Health, Faculty of Medicine Universitas Padjajaran—Dr. Hasan Sadikin General Hospital, Bandung, Indonesia; ^4^ Department of Physiology, Faculty of Medicine Universitas Indonesia, Jakarta, Indonesia; ^5^ Vrije Universiteit Brussel (VUB), UZ Brussel, KidZ Health Castle, Brussels, Belgium

**Keywords:** clinical findings, enteral feeding, feeding intolerance, scoring system, ultrasound, very preterm infants

## Abstract

Very preterm infants are at a high risk of developing feeding intolerance; however, there are no widely accepted definitions of feeding intolerance. This study aimed to develop a scoring system for feeding intolerance in very preterm infants by combining clinical symptoms and ultrasonography (US) findings. This prospective cohort study included very preterm and/or very low birth weight infants. We defined feeding intolerance as the inability to achieve full feeding (150 ml/kg/day) by 14 days of life. The clinical findings included vomiting, abdominal distention, and gastric fluid color. US findings included intestinal peristaltic frequency, gastric residual volume, peak systolic velocity, and the resistive index of the superior mesenteric artery. We conducted multivariate analyses to evaluate the potential predictors and developed a scoring system to predict feeding intolerance. A total of 156 infants fulfilled the eligibility criteria; however, 16 dropped out due to death. The proportion of patients with feeding intolerance was 60 (42.8%). Based on the predictive ability, predictors of feeding intolerance were determined using data from the US at 5–7 days of age. According to multivariate analysis, the final model consisted of 5 predictors: abdominal distention (score 1), hemorrhagic gastric fluid (score 2), intestinal peristaltic movement ≤18x/2 min (score 2), gastric fluid residue >25% (score 2), and resistive index >0.785 (score 2). A score equal to or above 5 indicated an increased risk of feeding intolerance with a positive predictive value of 84.4% (95% confidence interval:73.9–95.0) and a negative predictive value of 76.8% (95% confidence interval:68.4–85.3). The scoring system had good discrimination (area under the receiver operating characteristic curve:0.90) and calibration (*p* = 0.530) abilities. This study developed an objective, accurate, easy, and safe scoring system for predicting feeding intolerance based on clinical findings, 2D US, and color Doppler US.

## 1 Introduction

Very preterm infants have a higher risk of developing short- and long-term problems related to growth and development (1). They lose the golden period of accelerated growth in the third trimester; hence, an aggressive nutritional approach is necessary to prevent extrauterine growth retardation (2). However, providing adequate enteral nutrition can be challenging. Very preterm infants are at a higher risk of developing feeding intolerance; however, there are no widely accepted definitions of feeding intolerance in this population. Recent systematic reviews reported a broad range of definitions, from a single clinical sign to a combination of several clinical signs, timestamps, and qualitative judgment (3). Most neonatologists consider gastrointestinal dysmotility symptoms, such as increased gastric residue or abdominal distention, as feeding intolerance. However, these signs and symptoms can also be a physiological phenomenon due to gastrointestinal immaturity (4–6). This contradiction may lead to an overdiagnosis of feeding intolerance and cause a delay in the attainment of full enteral feeding (7).

Signs and symptoms of presumed feeding intolerance can also be found in necrotizing enterocolitis (NEC) (8). As a consequence, abdominal radiography is often performed to exclude NEC; however, it is not mandatory for infants with feeding intolerance. Radiography causes unnecessary radiation exposure in this vulnerable preterm population (9). Hence, a safe and accurate diagnostic tool is required to determine feeding intolerance. Ultrasonography (US) is a safe and dynamic imaging technique widely used in preterm infants. It can accurately measure the gastric content and volume, peristaltic movement, and intestinal blood flow (10, 11). Therefore, US has been proposed as a supporting examination for diagnosing feeding intolerance.

This study aimed to develop a scoring system for feeding intolerance in very preterm infants by combining clinical symptoms and US findings.

## 2 Materials and methods

This was a prospective cohort study of very preterm (28–32 weeks) and/or very low birth weight (VLBW) infants (<1,500 g) born at Cipto Mangunkusumo Hospital (CMH), a national tertiary referral hospital in Indonesia. This hospital has a level IV neonatal intensive care unit (NICU) with a multidisciplinary team that provides best practices in neonatal care. The study was conducted between March 2021 until August 2022. The Health Research Ethics Committee of the Faculty of Medicine at Universitas Indonesia approved this study (protocol number 21-01-0072). Informed consent was obtained from the parents or legal guardians of the infants.

The exclusion criteria included: infants with lethal and complex congenital disorders, such as gastroschisis, gastrointestinal atresia, congenital heart disease or heart syndrome; and those not able to undergo gastrointestinal US and superior mesenteric artery (SMA) color Doppler US within 48 h of birth due to clinical instability (e.g., desaturation or bradycardia due to US examination). Participants were considered to have dropped out if they died before the age of 14 days and had not yet achieved full feeding, or if they left against medical advice.

The primary outcome of this study was feeding intolerance. This was defined as the inability to achieve full feeding (150 ml/kg/day) by 14 days of age. The potential predictors of feeding intolerance were divided into clinical and US findings. Clinical findings included: vomiting (any vomiting in the last 24 h), abdominal distention (per attending discretion), and gastric fluid color (clear, hemorrhagic, or bile stained). For gastric fluid color, we observed the gastric fluid that come out from feeding tube, not from gastric aspiration. US findings included: intestinal peristaltic frequency, gastric residual volume (GRV), peak systolic velocity (PSV), and resistive index (RI).

Data regarding antenatal history, birth history, and clinical data at birth were acquired from medical records. Maternal data consisted of age, history of antenatal care (ANC), urinary tract infection (UTI), preterm rupture of membranes (PROM), absent or reverse end-diastolic flow (AREDF), history of preeclampsia/eclampsia, and history of prenatal magnesium sulfate or steroid administration. The infants' data consisted of sex, gestational age, birth weight, APGAR score at 5 min of life, active resuscitation at birth, and history of surfactant administration. Clinical data included the use of respiratory support [high-flow nasal cannula, continuous positive air pressure (CPAP), invasive mechanical ventilation, or high-frequency oscillatory ventilation] and the highest oxygen fraction. Clinical data, gastrointestinal symptoms (vomiting, abdominal distension, and gastric fluid discoloration), and comorbidities were documented daily for the first 14 days after birth. The algorithm of preterm infant feeding in our unit can be seen in Supplementary Figure S1.

The infants underwent both two-dimensional (2D) and color Doppler US at the age of 48 h or less, 7 days, and 14 days or when full feeding was achieved. If the infants had received feeding of 10 ml/kg/day or more, the examination was performed before and 60 min after feeding. If the infants had not received feeding of 10 ml/kg/day or more, the examination was only performed once before feeding. Examination of intestinal peristaltic movement was performed in four abdominal quadrants for 30 s each. Peristaltic frequency was calculated as the sum of all peristaltic movements in the four quadrants. The examination for GRV was performed in longitudinal and transversal view and the volume was calculated automatically by the US machine (Phillips Affiniti 50s). The results are expressed as gastric residue percentages, calculated as follows: (GRV/previous feeding volumes) × 100.

Color Doppler US of the SMA was performed to assess the PSV and end-diastolic velocity (EDV). The formula used to calculate the RI was (PSV — EDV)/PSV, which was performed automatically by the US machine.

The ultrasound operators (*n* = 2) had good intra-and inter-observer reliabilities (kappa = 0.932–0.996). To avoid observer bias, the operators were blinded to the infants' clinical status. All US parameters were measured objectively by automatic calculations from the US machine; hence, we could not manipulate the US results. Moreover, we did not know the cutoff point of each US parameter, which was the objective of the study. Attending neonatologists were blinded to the US results.

### 2.1 Statistical analysis

Normally distributed continuous data are presented as means and standard deviations, and non-normally distributed continuous data are presented as medians and ranges. Categorical data are presented as proportions. Parametric tests for continuous data were performed using independent *t*-tests, whereas non-parametric tests were performed using the Mann-Whitney *U*-test. Categorical data were analyzed using the chi-square or Fisher's exact test.

For the US parameters (intestinal peristaltic frequency, GRV, PSV, and RI), we generated a receiver operating characteristic (ROC) curve and calculated the area under the curve (AUC) to evaluate its predictive ability and set the optimal cut-off based on the best combination of sensitivity and specificity. A variable with an AUC above 0.60 was considered to have a good discrimination ability. All US parameters were dichotomized based on the optimal cutoff before proceeding to bivariate analysis.

We conducted both bivariate and multivariate analyses to evaluate the potential predictors of feeding intolerance using multiple logistic regression. Effect size estimates were calculated using odds ratios (ORs) and 95% confidence intervals (CIs). Variables with a *p*-value of less than 0.20 in the bivariate analysis were included in model development. A stepwise approach was used to develop a predictive model of feeding intolerance. All variables with a *p*-value of less than 0.05 were considered significant predictors and were retained in the model. Other variables that changed the OR by more than 10% were considered confounders and were included in the model, although they were not statistically significant. Each variable in the final model was assigned a score based on its standardized coefficient (regression coefficient divided by the standard error). To make the score easier to use, each standardized coefficient from the variables in the final model was divided by the smallest standardized coefficient among them and rounded to the nearest integer. Discrimination and calibration evaluations were performed using AUC analysis from the ROC curve and the Hosmer-Lemeshow test. A model with an AUC above 0.60 was considered to have good discrimination, while a *p*-value more than 0.05 from the Hosmer-Lemeshow test was considered good calibration. The cutoff points were determined based on the best combination of sensitivity, specificity, positive predictive value (PPV), and negative predictive value (NPV). Data analyses were performed using SPSS version 20.0 (Armonk, NY: IBM Corp).

## 3 Results

**Figure 1:**
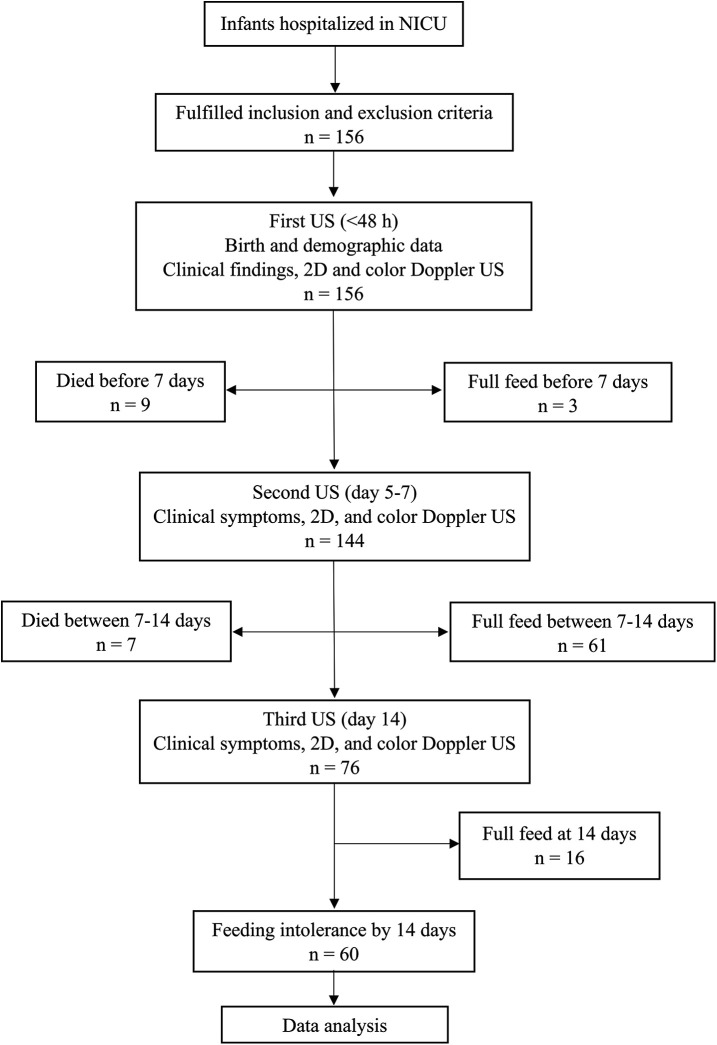
Flowchart of the study.

A total of 156 infants met the eligibility criteria. Sixteen infants dropped out: nine died before the age of 7 days and 7 died between 7 and 14 days of age, before reaching full feeding. Hence, a total of 140 infants were included in this study. Among them, 60 (42.8%) developed feeding intolerance (Figure 1).

Maternal and infant characteristics are shown in Tables 1, 2, respectively. The mean maternal age was 29.7 (±6) years. Among these, 38.5% had PROM, 25.0% had preeclampsia, and 19.9% had AREDF. A total of 86.5% of infants were born between 28 and 32 weeks with a mean birthweight of 1,278 (±275) g. Based on their comorbidities, 85.9% of infants had respiratory distress syndrome (RDS), and 51.9% received surfactant treatment. The proportions of patients with hemodynamically significant patent ductus arteriosus (hs-PDA), intraventricular hemorrhage (IVH), and sepsis were 34%, 31.4%, and 16%, respectively. Among all infants, 16 (11.4%) were developed NEC (stage II or more). It was found mostly among infants with feeding intolerance (25%) compared to no feeding intolerance (1.3%).

**Table 1:** Characteristics of the subjects’ mothers.

Characteristics	*N* = 156
Age	
Mean, (SD)	29.7 (6.0)
Median, (min—max)	30 (16–42)
History of antenatal care, *n* (%)	
Irregular visit	12 (7.7%)
Regular visit	144 (92.3%)
Preterm rupture of membranes, *n* (%)	
Yes	60 (38.5%)
No	96 (61.5%)
Urinary tract infection, *n* (%)	
Yes	19 (12.2%)
No	137 (87.8%)
Placental blood flow, *n* (%)	
Absent/reserve	31 (19.9%)
Normal	79 (50.6%)
Not examined	46 (29.5%)
History of preeclampsia or eclampsia, *n* (%)	
Yes	39 (25.0%)
No	117 (75.0%)
History of MgSO_4_ therapy, *n* (%)	
Yes	17 (10.9%)
No	139 (89.1%)
Parity, *n* (%)	
Nulliparous	63 (40.4%)
Multiparous	93 (59.6%)
Mode of delivery, *n* (%)	
Caesarean section	123 (78.8%)
Vaginal delivery	33 (21.2%)
History of antenatal steroid use, *n* (%)	
Yes	75 (48.1%)
No	81 (51.9%)

**Table 2:** Characteristics of the study subjects.

Infant's characteristics	*n* (%)
Sex	
Male	72 (46.2%)
Female	84 (53.8%)
Gestational age	
Mean (SD)	30.35 (1.93)
Median (min—max)	30 (26–38)
<28 weeks	5 (3.2%)
28 – <32 weeks	135 (86.5%)
≥32 weeks	16 (10.3%)
Birth Weight	
Mean (SD)	1,278.30 (275.3)
Median (min—max)	1,295 (650–2,080)
<1,000 g	22 (14.1%)
1,000 – <1,500 g	104 (66.7%)
>1,500 g	30 (19.2%)
APGAR Score at 5 min of life	
0–3	1 (0.6%)
4–6	43 (27.6%)
7–10	112 (71.8%)
Surfactant	
Present	81 (51.9%)
None	75 (48.1%)
Active resuscitation at birth	
None	3
CPAP	121
PPV	76
Intubation	58

The proportion of infants who received enteral feeding was 21.2% in the first US, 63.9% in the second, and 65.7% in the third. In the first US, 14.1% of infants had abdominal distension. This proportion was consistent between the second and third US examinations. A total of 7.1% of the infants vomited in the last 24 h at the first US, which did not increase through the second and third US. In the first US, 34.0% and 1.3% of the infants had hemorrhagic and bile-stained gastric fluid color, respectively. This decreased to 21.2% and 0.7% in the second US and to 9.4% and 3.1% in the third US, respectively. Detailed information on the clinical findings on the day of US is shown in Supplementary Table S1.

In the first US, the mean percentage of infants gastric fluid residue volume was 70 (±35) %, which reduced to 55 (±29)% in the second US and 46 (±24)% in the third US. The AUC of gastric fluid residue volume was poor in the first US (0.538), but improved in the second and third US, with an AUC of 0.713 (95% CI: 0.629–0.797) and 0.602 (95% CI: 0.508–0.696), respectively. The intestinal peristaltic frequency in the first US was 15.6 (±9.4), increasing to 21.7 (±9.8) and 19.2 (±14.8) in the second and third US, respectively. Similarly, the intestinal peristaltic frequency in the first US showed poor discrimination of feeding intolerance (AUC: 0.551; 95% CI: 0.455–0.647). In the second and third US, the discrimination was good, with an AUC of 0.797 (95% CI: 0.725–0.869) and 0.882 (95% CI: 0.826–0.938), respectively. Detailed information on the 2D US findings is provided in Supplementary Table S2.

The ability of color Doppler US parameters to predict feeding intolerance varied between the US time points. The AUC at the first US of PSV and RI were poor (0.552 [95% CI: 0.456–0.648] and 0.551 [95% CI: 0.455–0.647], respectively). The AUC for PSV and RI increased slightly in the second US (0.572 [95% CI: 0.477–0.667] and 0.642 [95% CI: 0.551–0.733], respectively) and third US (0.558 [95% CI: 0.462–0.654] and 0.634 [95% 0.543–0.725], respectively). Detailed information on the color Doppler US findings is provided in Supplementary Table S3.

Based on these findings, the data from the first and third US had low discrimination ability (AUC < 0.60), whereas the second US showed good discrimination ability. Therefore, the next analysis to find the predictors for feeding intolerance were derived from the second US. We only use pre-feeding US data for the scoring system construction. The optimal cut-off points for US parameters were GRV more than 25%, an intestinal peristaltic frequency of less than or equal to 18 times per 2 min, a PSV of 128.5 cm/s or less, and an RI of more than 0.785.

Table 3 presents the results of the univariate analysis. PSV, bile-stained gastric fluid, and vomiting were not included in the multivariate analysis because of *p*-values more than 0.20.

**Table 3:** The predictor candidates based on the feeding intolerance status at the second US.

Predictors	Feeding status				RR (95% CI)	*P*-value
	Intolerance		Tolerance			
	*n*	%	*n*	%		
PSV						
≤128.5	42	41.6	59	58.4	0.90 (0.59–1.36)	0.624
>128.5	18	46.2	21	53.8		
RI						
>0.785	49	50.5	48	49.5	1.97 (1.14–3.41)	0.006
≤0.785	11	25.6	32	74.4		
Residual percentage						
>25.625	54	60.7	35	39.3	5.16 (2.39–11.14)	<0.001
≤25.625	6	11.8	45	88.2		
Peristaltic volume						
≤18	31	75.6	10	24.4	2.58 (1.82–3.67)	<0.001
>18	29	29.3	70	70.7		
Hemorrhagic gastric fluid						
Yes	23	92.0	2	8.0	2.86 (2.14–3.82)	<0.001
No	37	32.2	78	67.8		
Bile-stained gastric fluid						
Yes	1	100.0	0	0.0	2.36 (1.94–2.86)	0.429
No	59	42.4	80	57.6		
Vomiting						
Yes	4	36.4	7	63.6	0.84 (0.37–1.87)	0.758
No	56	43.4	73	56.6		
Abdominal distension						
Yes	17	70.8	7	29.2	1.91 (1.35–2.71)	0.002
No	43	37.1	73	62.9		

According to the multivariate analysis, the final model consisted of five predictors (Table 4). The scoring system is listed in Table 5. The final model had good discrimination (AUC: 0.90; 95% CI: 0.849–0.951) and calibration (*p* = 0.530) abilities. A score of 5 or more showed an increased risk of feeding intolerance; hence, feeding should be stopped (PPV: 84.4% [95% CI: 73.8%–95.0%] and NPV: 76.8% [95% CI: 68.4%–85.3%]). A score of 3 or less showed a low risk of feeding intolerance; hence, enteral feeding should be continued [PPV: 65.2% (95% CI: 55.3%–75.1%) and NPV: 96.10% (95% CI; 90.7%–100%)]. A score of 4 was a gray area in which the feeding volume should not be increased and the score should be re-evaluated at the next feeding schedule within 24 h. Any changes in the score during re-evaluation would be interpreted according to the same scoring system.

**Table 4:** The final model of multivariate analysis of the predictors for feeding intolerance.

Predictors	Coefficient	OR	95% CI	*P*-value
RI >0.785	1.995	7.350	2.055–26.292	0.002
Gastric fluid residue >25.6%	2.062	7.858	2.420–25.514	0.001
Intestinal peristaltic movement ≤18 × /2 min	2.095	8.126	2.737–24.122	<0.001
Abdominal distension	1.116	3.053	0.855–10.898	0.086
Hemorrhagic gastric fluid color	3.165	23.861	3.928–142.783	0.001

**Table 5:** The feeding intolerance prediction scoring system for very preterm and VLBW infants.

Predictors	Score
RI >0.785	2
GRV >25%	2
Intestinal peristaltic frequency ≤18 × /2 min	2
Hemorrhagic gastric fluid color	2
Abdominal distension	1
Interpretation Score ≥ 5: stop feeding Score 4: continued feeding without volume advancement and a re-evaluation in the next feeding schedule in 24 h Score ≤ 3: continue feeding	
Notes: 1. US and clinical examinations are performed at the age of 5–7 days. 2. Clinical assessment (hemorrhagic gastric fluid color and abdominal distension) is observed within the last 24 h 3. Formula for gastric residual volume percentage: $\frac{\mathrm{Gastric}\;\mathrm{residual}\;\mathrm{volume}}{\mathrm{Previous}\;\mathrm{feeding}\;\mathrm{volume}}\times \;100$	

## 4 Discussion

To the best of our knowledge, this is the first cohort study to predict feeding intolerance in very preterm and/or VLBW infants by combining clinical and US findings. We found that abdominal distension, hemorrhagic gastric fluid color, RI more than 0.785, GRV more than 25%, and intestinal peristaltic movement of less than or equal to 18 times in 2 min at 5–7 days of age are independent predictors of feeding intolerance in very preterm or VLBW infants. We developed the first scoring system to predict feeding intolerance that utilizes US. These findings may be invaluable for improving the nutritional care of preterm and VLBW infants.

The widely agreed definition of feeding intolerance is the “inability to digest enteral food presented as a GRV of more than 50%, abdominal distension or emesis or both, and disruption of the patient's feeding plan” (12). However, the presence of either high GRV, abdominal distension, or emesis as a predictor of feeding intolerance has been under-investigated.

There are two parameters to assess GRV: an absolute parameter (GRV >2–5 ml/kg) and a relative parameter [expressed as a percentage (%) of residue from the previous feeding volume] (13). Previous studies mostly used a relative GRV of more than 20%–30% (14, 15) as an indicator of feeding intolerance; however, recent literature uses a cutoff of more than 50% (16, 17). In clinical practice, GRV influences the feeding schedule; hence, it is more suitable as an independent variable for the development of feeding intolerance than as an indicator for feeding intolerance diagnosis (13).

GRV is conventionally measured by aspiration of gastric fluid. Gastric aspiration has advantages and disadvantages in preterm neonates (18). Besides being invasive, routine GRV measurements to adjust the feeding program lengthen the duration of full feeding and hospital stay (19). A randomized controlled trial by Singh et al. of 87 preterm infants weighing between 1,500 and 2,000 g also showed that routine gastric residue aspiration did not accelerate the time to reach full feeding compared with only evaluating hemorrhagic gastric fluid, emesis, and abdominal distention (20). In our scoring system, we recommend clinician do a visual observation of gastric fluid color instead of routine gastric aspiration.

The number of infants with vomiting increased from the first to the third US visit. In daily clinical practice, a preterm baby with vomiting is frequently subjected to abdominal x-ray examination, which is unnecessary and harmful to an infant susceptible to radiation. Vomiting along with other clinical symptoms may be a sign of feeding intolerance (5). Otherwise, vomiting without any other symptoms should not be used as a guide for further examination (2, 9, 10). This is following the data of this study showing that vomiting was not a predictor for feeding intolerance.

Abdominal distention as a predictor of feeding intolerance has not been extensively studied. A previous study showed that abdominal distention has a poor predictive value for feeding outcomes in preterm infants (21, 22). Hence, clinicians should specify the characteristics of abdominal distention and the presence of other symptoms. Local and systemic signs may increase the likelihood of feeding intolerance. Local signs include the absence of bowel sounds, abdominal discoloration, and bloody stools, while systemic signs include apnea, bradycardia, and temperature instability (13). In this study, abdominal distention was combined with other findings to improve the predictive value of the scoring system.

Hemorrhagic gastric fluid was a predictor of feeding intolerance, whereas bile-stained gastric fluid was not. Several studies have reported that feeding should not be delayed if the gastric fluid is bile-stained. Bile-stained gastric fluid can be caused by bile acid reflux during prematurity, sepsis, or transpyloric feeding tube placement (11–13). Hemorrhagic gastric fluid color can be caused by traumatic damage (feeding tube placement, upper airway suction, and non-invasive respiratory support), stress-induced mucosal lesions, loss of mucosal integrity, abnormal bacterial colonization, and compromised intestinal blood flow. Compromised intestinal blood flow can also be detected by evaluating SMA flow. Although the role of hemorrhagic gastric fluid color in the management of a feeding plan is unclear, its combination with other parameters, such as SMA flow, may increase its predictive ability.

The major novelty of this study is the use of US to evaluate GRV and SMA blood flow parameters in predicting feeding intolerance. We found that GRV (measured using US) along with abdominal distention and hemorrhagic gastric fluid were independent predictors of feeding intolerance. GRV measurement using US has good validity and reproducibility (10, 11).

Studies on SMA blood flow have been an interesting topic in recent years. When evaluating SMA blood flow, both PSV and RI are common US indicators being used. In this study, we found that the RI was an independent predictor of feeding intolerance. Previous studies have reported conflicting results regarding the ability of SMA blood flow to predict feeding intolerance. Jain et al. did not find any correlation between SMA blood flow using PSV, EDV, time-averaged mean velocity, and RI to feeding intolerance; however, they only recruited a small sample of VLBW infants (23). In contrast, Fang et al. found that RI had a moderate positive correlation with duration to reach full feeding (*r* = 0.6) as well as mean velocity (*r* = −0.49) at 60 min after test feeding (0.5 ml) (24). An SMA blood flow increase of more than 17% after feeding increases the success of full feeding with a sensitivity of 100% and specificity of 70% (24).

Our scoring system was constructed using clinical and US findings between 5 and 7 days of life. The risk of feeding intolerance was: 85% if the score was 5 or more, 23% if the score was 4 or less, and 5% if the score was 3 or less. Our final scoring system classified infants into low risk (score ≤ 3), intermediate risk (score 4), and high risk (score ≥ 5). Hence, a score of 5 or more was recommended as a cut-off to stop feeding, while a score of 3 or less was a cut-off to continue feeding. For infants with a score of 4, we recommend continued feeding without volume advancement and a re-evaluation in the next feeding schedule in 24 h. The next approach after the recommendation to stop feeding will depend on each unit's clinical pathway. Otherwise, we recommend the clinician do a thorough examination to find the possible cause of feeding intolerance such as infection, any GI abnormalities, use of medication that reduces gut motility (e.g., morphine), or hypoxia related to several conditions (PDA, respiratory problems).

Several literatures state that feeding intolerance is an initial presentation of NEC (3, 13, 25). Therefore, the presence of feeding intolerance symptoms is often misinterpreted as an early sign of NEC, so clinicians often stop feeding. In this study, it was found that not all infants with feeding intolerance had NEC (only 25%). Stopping feeding to non-feeding intolerant infants would be very detrimental. This scoring system can be used to determine if feeding intolerance is suspected and whether feeding needs to be stopped or not. Whether this score can be used to predict NEC cannot be explained from this study, thus, further research is needed.

There were some limitations in this study. The proportion of infants who received feeding at the first 48 h is low, thus we were unable to evaluate the gut motility and changes in SMA flow using US on those infants. The utility of this scoring system outside of the 5–7 days also needs to be further studied. The specific impact of some conditions such as NEC, central line-associated bloodstream infection (CLABSI), prolonged TPN, hs-PDA, and sepsis should be further studied to complete the understanding of feeding intolerance among preterm infants. An external validation study should be the next step to evaluate the generalizability use of this scoring system.

The scoring system developed in this study, which combines clinical and US findings, can be used to predict feeding intolerance in very preterm and/or VLBW infants. This may help clinicians plan feeding programs for infants at risk of feeding intolerance and prevent complications of long-term parenteral nutrition. This scoring system was safe and easy to use. Further study about US utilization in evaluating feeding intolerance among extremely preterm and extremely low birth weight infants is necessary.

## Data Availability

The raw data supporting the conclusions of this article will be made available by the authors, without undue reservation.
