# Supplementary material for: Feeding intolerance scoring system in very preterm and very low birth weight infants using clinical and ultrasound findings
Source: Front Pediatr. 2024 Apr 25;12:1370361. doi: 10.3389/fped.2024.1370361 (PMC11079181; doi:10.3389/fped.2024.1370361)
Supplement: Supplementary file 1 [file Datasheet1.pdf]

Supplementary figure 1

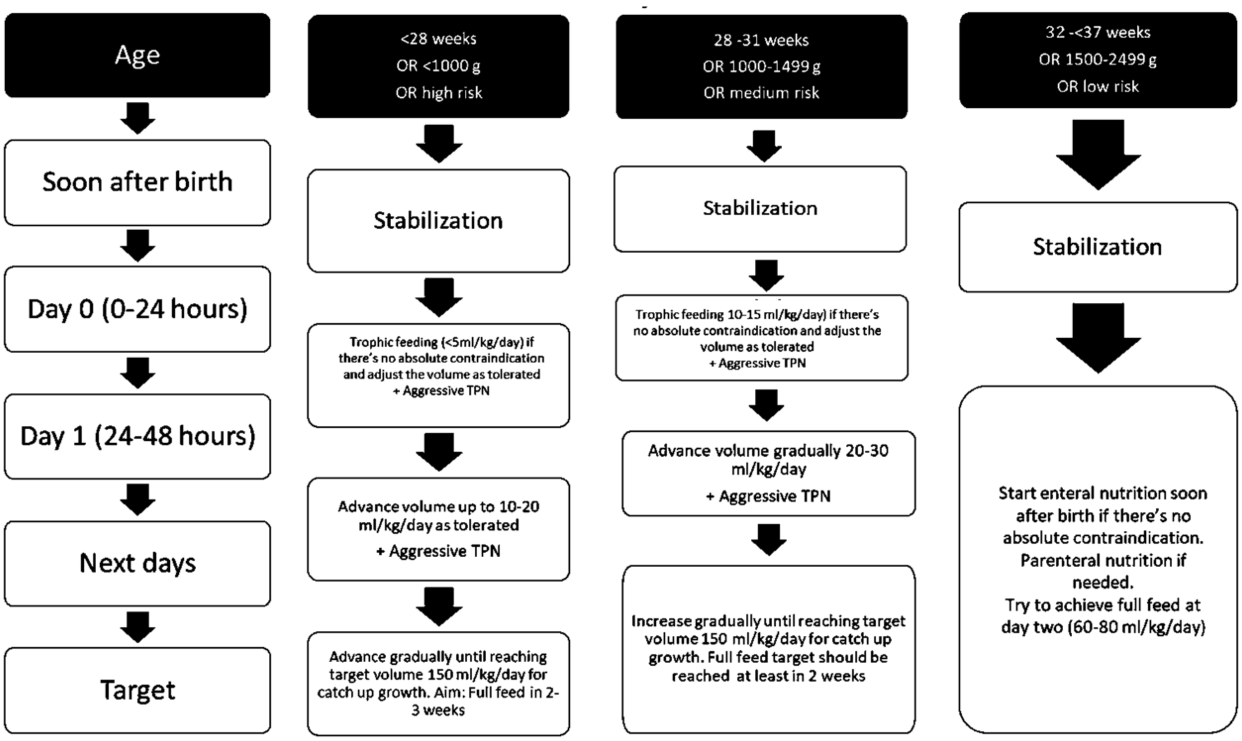

Supplementary table 1

Parameter	1st US	2nd US	3rd US
Abdominal distension*			
No	134 (85,9%)	121 (82,3%)	107 (83,6%)
Yes, not tense	19 (12,2%)	25 (17,0%)	20 (15,6%)
Yes, tense	3 (1,9%)	1 (0,7%)	1 (0,8%)
Vomiting			
No	145 (92.9%)	136 (87,2%)	116 (90,6%)
Yes	11 (7,1%)	11 (7,1%)	12 (9,4%)
Gastric fluid color			
Clear or white (milk)	101 (64,7%)	115 (78,2%)	112 (87,5%)
Hemorrhagic gastric fluid	53 (34,0%)	31 (21,1%)	12 (9,4%)
Bile-stained gastric fluid	2 (1,3%)	1 (0,7%)	4 (3,1%)

*In the analysis, we combine tense and not tense abdominal distention into a category of abdominal distention

Supplementary Table 2

**2D US Parameter**	**1st US**	**2nd US**	**3rd US**
Gastric residual volume before feeding	2.08 ±2.50	3.26 ±3.12	3.16 ±2.95
Gastric residual volume after feeding*	3.41 ±2.60	8.15 ±6.03	9.37 ±5.82
Gastric residual fluid (%)	70.2 ±35.0	55.1 ±29.2	46.5 ±24.5
Intestinal peristaltic frequency	15.6 ±9.4	21.7 ±9.8	19.2 ±14.8
Received enteral feeding	45 (28.8%)	101 (68.7%)	103 (80.5)

*Only calculated if infants had received feeding in the 1st US (n = 45), 2nd US (n = 101), and 3rd US (n = 103)

Supplementary table 3

**Color Flow Doppler US Parameter**	**1st US**	**2nd US**	**3rd US**
**Before feeding**	n=156	n=147	n=128
PSV (mean±SD)	90.0 ±36.4	110.2 ±37.7	112.5 ±30.9
EDV (mean±SD)	19.4 ± 13.2	23.1 ±8.7	21.4 ±7.9
RI (mean±SD)	0.78 ±0.10	0.78 ±0.07	0.80 ±0.07
**After feeding**	n=45	n=101	n=103
PSV (mean±SD)	110.7 ±37.6	137.7±50.5	155.9 ±50.3
EDV (mean±SD)	22.6 ±9.9	25.2 ±12.0	26.3 ±9.3
RI (mean±SD)	0.77 ±0.08	0.81 ±0.07	0.83 ±0.05
